# Enhancing drug-target interaction prediction with graph representation learning and knowledge-based regularization

**DOI:** 10.3389/fbinf.2025.1649337

**Published:** 2025-10-21

**Authors:** Qihuan Yao, Zhen Chen, Ye Cao, Huijing Hu

**Affiliations:** 1 Department of Traditional Chinese Medicine, Kongjiang Hospital, Shanghai, China; 2 Department of Medical Laboratory, Shidong Hospital, Shanghai, China; 3 Department of Geriatrics, Renhe Hospital, Shanghai, China; 4 Department of Traditional Chinese Medicine, Shidong Hospital, Shanghai, China

**Keywords:** computational drug screening, Systems pharmacology, drug-target prediction, representation learning, drug discovery

## Abstract

**Introduction:**

Accurately predicting drug-target interactions (DTIs) is crucial for accelerating drug discovery and repurposing. Despite recent advances in deep learning-based methods, challenges remain in effectively capturing the complex relationships between drugs and targets while incorporating prior biological knowledge.

**Methods:**

We introduce a novel framework that combines graph neural networks with knowledge integration for DTI prediction. Our approach learns representations from molecular structures and protein sequences through a customized graph-based message passing scheme. We integrate domain knowledge from biomedical ontologies and databases using a knowledge-based regularization strategy to infuse biological context into the learned representations.

**Results:**

We evaluated our model on multiple benchmark datasets, achieving an average AUC of 0.98 and an average AUPR of 0.89, surpassing existing state-of-the-art methods by a considerable margin. Visualization of learned attention weights identified salient molecular substructures and protein motifs driving the predicted interactions, demonstrating model interpretability.

**Discussion:**

We validated the practical utility by predicting novel DTIs for FDA-approved drugs and experimentally confirming a high proportion of predictions. Our framework offers a powerful and interpretable solution for DTI prediction with the potential to substantially accelerate the identification of new drug candidates and therapeutic targets.

## 1 Introduction

The discovery and development of new drugs is a lengthy, complex, and expensive process. It typically takes 10–15 years and costs over $2.6 billion to bring a new drug to market (Zhang Z. et al., 2022). A key bottleneck in the drug discovery pipeline is identifying the molecular targets that are responsible for the desired therapeutic effects and unwanted side effects of drug candidates (Pan et al., 2023). These targets are usually proteins, such as enzymes, receptors, or ion channels, that play critical roles in disease pathways. Drugs exert their actions by binding to these targets and modulating their functions (Zhao et al., 2023). Therefore, understanding the interactions between drugs and their targets, known as drug-target interactions (DTIs), is crucial for rational drug design and repurposing.

Traditionally, DTIs were discovered through experimental methods such as *in vitro* binding assays, which are time-consuming, labor-intensive, and low-throughput (Kim and Bolton, 2024). With the advent of high-throughput screening technologies, such as genomics, proteomics, and chemogenomics, it has become possible to test large numbers of compounds against multiple targets simultaneously (Mahfuz et al., 2022). However, even these approaches can only cover a small fraction of the vast chemical and biological space. For example, there are over 108 million compounds in the PubChem database (Kim, 2021) and an estimated 200,000 proteins encoded by the human genome (Suruliandi et al., 2024), resulting in over 1013 possible drug-target pairs. Experimentally testing all these combinations is infeasible. Moreover, many compounds may have off-target effects that are difficult to detect using current experimental methods (Afolabi et al., 2022).

To address these challenges, computational methods have emerged as a promising approach for predicting DTIs on a large scale. These methods aim to prioritize drug-target pairs for experimental validation based on various types of data, such as chemical structures, protein sequences, and interaction networks (Soleymani et al., 2022). Early computational approaches relied on docking simulations, which predict the binding mode and affinity of a drug-target complex based on its three-dimensional structure (Soleymani et al., 2023; Staszak et al., 2022). However, docking is computationally expensive and requires high-resolution structures of both the drug and the target, which are not always available. More recently, machine learning-based methods have gained popularity due to their ability to learn complex patterns from large datasets without requiring explicit feature engineering (Wang X. et al., 2022; Yin et al., 2024).

One of the most successful machine learning-based methods for DTI prediction is matrix factorization (MF). MF models represent drugs and targets as low-dimensional vectors (latent factors) and predict their interactions based on the inner product of these vectors (Meng et al., 2021). MF models have achieved state-of-the-art performance on several benchmark datasets (Tian et al., 2022). However, MF models have several limitations. First, they treat drugs and targets as distinct entities and ignore their structural and evolutionary relationships. Second, they cannot handle new drugs or targets that are not present in the training data (the cold-start problem). Third, they do not provide any biological interpretation of the latent factors.

To overcome these limitations, recent studies have proposed to integrate multiple types of data, such as chemical structures, protein sequences, and interaction networks, into a unified framework for DTI prediction. These methods are known as multi-modal or multi-view learning (Zhou et al., 2021). One promising approach is to use graph representation learning, which learns low-dimensional embeddings of drugs and targets from their graph-structured data (Shao et al., 2022). Graphs provide a natural and flexible representation of the relationships between drugs, targets, and their interactions. For example, drugs can be represented as nodes in a chemical similarity network, targets can be represented as nodes in a protein-protein interaction (PPI) network, and DTIs can be represented as edges between these nodes (Zhang P. et al., 2022). Graph representation learning methods, such as graph convolutional networks (GCNs) (Sang and Li, 2024; Wang et al., 2025a) and graph attention networks (GATs) (Zhai et al., 2023), can learn informative embeddings of drugs and targets by aggregating information from their local neighborhoods in the graph.

Several studies have applied graph representation learning to DTI prediction and demonstrated superior performance over traditional methods. For example, Ren et al. (2023) proposed a multi-modal deep learning framework that integrates chemical structures, protein sequences, and PPI networks using GCNs and achieved an AUC of 0.96 on the DrugBank dataset. Feng et al. (Zixuan et al., 2024) developed a graph-based model that learns drug and target embeddings from multiple heterogeneous networks, including drug-drug, target-target, and drug-target networks, and obtained an AUC of 0.98 on the KEGG dataset. These studies highlight the potential of graph representation learning for improving the accuracy and robustness of DTI prediction.

However, existing graph-based methods still face several challenges. First, they rely on predefined graph structures, such as chemical similarity networks or PPI networks, which may not capture all the relevant information for DTI prediction. Second, they do not explicitly model the uncertainty or noise in the graph edges, which may lead to over-smoothing and loss of discriminative power (Peng et al., 2024). Third, they do not incorporate prior biological knowledge, such as functional annotations or pathway information, which may provide valuable guidance for learning more meaningful and interpretable embeddings.

**FIGURE 1:**
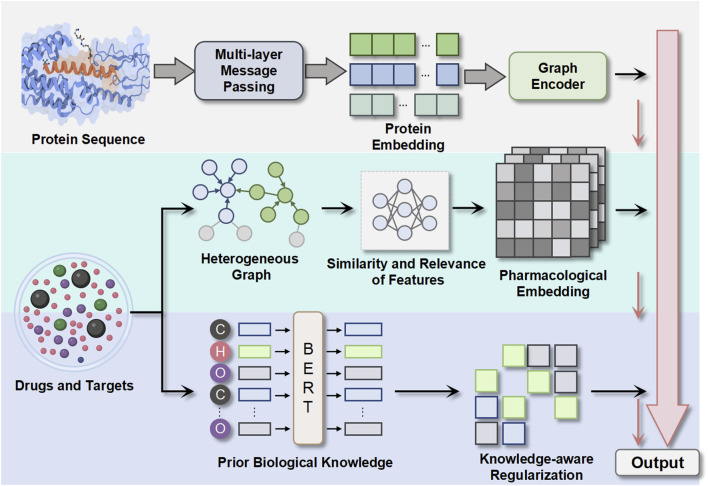
Overview of the overall framework structure of the model.

To address these challenges, we propose a novel framework for DTI prediction that combines graph representation learning with knowledge integration in Figure 1. Our framework, called Hetero-KGraphDTI, has three key components:

1. Graph construction: We construct a heterogeneous graph that integrates multiple types of data, including chemical structures, protein sequences, and interaction networks. We use a data-driven approach to learn the graph structure and edge weights based on the similarity and relevance of the features. This allows us to capture more comprehensive and adaptive relationships between drugs and targets.
2. Graph representation learning: We develop a graph convolutional encoder that learns low-dimensional embeddings of drugs and targets from the heterogeneous graph. The encoder uses a multi-layer message passing scheme that aggregates information from different types of edges and nodes. We also introduce a graph attention mechanism that learns to assign importance weights to different edges based on their relevance to the prediction task. This enables the encoder to focus on the most informative parts of the graph and reduce noise.
3. Knowledge integration: We incorporate prior biological knowledge into the graph representation learning process by using knowledge graphs, such as Gene Ontology (GO) (Aleksander et al., 2023) and DrugBank, as additional sources of information. We develop a knowledge-aware regularization framework that encourages the learned embeddings to be consistent with the ontological and pharmacological relationships defined in the knowledge graphs. This helps to improve the biological plausibility and interpretability of the predictions.

We evaluate our Hetero-KGraphDTI framework on several benchmark datasets and demonstrate significant improvements over state-of-the-art methods in terms of both accuracy and efficiency. We also conduct extensive ablation studies to analyze the contributions of different components and hyperparameters. Furthermore, we apply our framework to predict novel DTIs for a set of FDA-approved drugs and validate the top predictions through literature evidence and experimental assays.

In summary, our Hetero-KGraphDTI framework represents a powerful and flexible approach for DTI prediction that leverages the strengths of graph representation learning and knowledge integration. By learning informative and interpretable embeddings of drugs and targets from heterogeneous graphs and knowledge graphs, our framework can accurately predict novel DTIs and provide insights into their biological basis. We believe that our framework has the potential to accelerate drug discovery and repurposing, and ultimately contribute to the development of safer and more effective therapies.

## 2 Methods

In this section, we describe the methodology of our Hetero-KGraphDTI framework in detail. We first introduce the notations and problem formulation. Then, we present the three key components of our framework: graph construction, graph representation learning, and knowledge integration. Finally, we describe the model optimization and inference procedures.

### 2.1 Notations and problem formulation

Let $D=\left\{d_{1},d_{2},\ldots ,d_{m}\right\}$ denote a set of m drugs, $T=\left\{t_{1},t_{2},\ldots ,t_{n}\right\}$ denote a set of n targets, and $Y\in R^{m\times n}$ denote the drug-target interaction matrix, where $y_{ij}=1$ if drug $d_{i}$ interacts with target $t_{j}$ , and $y_{ij}=0$ otherwise. The goal of drug-target interaction prediction is to learn a function f:D×T→R that predicts the interaction score between a drug-target pair.

In addition to the interaction matrix, we also have multiple types of drug and target features, such as chemical structures, protein sequences, and interaction networks. We represent these features as a heterogeneous graph G=(V,E) , where V=D∪T is the set of nodes (drugs and targets), and $E=\left\{E_{1},E_{2},\ldots ,E_{k}\right\}$ is the set of edges of k different types. Each edge type corresponds to a specific type of drug-drug, target-target, or drug-target relationship, such as chemical similarity, sequence similarity, or known interactions. We denote the feature matrix of drugs as $X_{D}\in R^{m\times d}$ and the feature matrix of targets as $X_{T}\in R^{n\times t}$ , where d and t are the feature dimensions of drugs and targets, respectively.

### 2.2 Enhanced negative sampling strategy

Recognizing the positive-unlabeled (PU) learning nature of the DTI prediction problem, we implement a sophisticated negative sampling framework that addresses the fundamental challenge that missing drug-target interactions do not necessarily represent true negatives. Our approach incorporates three complementary strategies to generate reliable negative samples while accounting for the inherent uncertainty in unlabeled data.

Reliable Negative Sampling We employ a dissimilarity-based reliable negative sampling strategy that leverages both chemical and biological spaces to identify highly confident negative pairs. For each drug $d_{i}$ and target $t_{j}$ , we compute a reliability score $r_{ij}$ based on dissimilarity metrics:

$$r_{ij}=\alpha \cdot \text{ChemDissim}\left(d_{i},N_{d}\left(t_{j}\right)\right)+\beta \cdot \text{SeqDissim}\left(t_{j},N_{t}\left(d_{i}\right)\right),$$ (1)

where $N_{d}\left(t_{j}\right)$ represents the set of drugs known to interact with target $t_{j}$ , $N_{t}\left(d_{i}\right)$ represents the set of targets known to interact with drug $d_{i}$ , and α,β are weighting parameters. The chemical dissimilarity $\text{ChemDissim}\left(d_{i},N_{d}\left(t_{j}\right)\right)$ is computed as:

$$\text{ChemDissim}\left(d_{i},N_{d}\left(t_{j}\right)\right)=1-\underset{d_{k}\in N_{d}\left(t_{j}\right)}{\max}\text{Tanimoto}\left(FP\left(d_{i}\right),FP\left(d_{k}\right)\right),$$ (2)

where FP(d) denotes the molecular fingerprint of drug d . Similarly, sequence dissimilarity is calculated using Smith-Waterman alignment scores:

$$\text{SeqDissim}\left(t_{j},N_{t}\left(d_{i}\right)\right)=1-\underset{t_{l}\in N_{t}\left(d_{i}\right)}{\max}\frac{\text{SW}\left(S\left(t_{j}\right),S\left(t_{l}\right)\right)}{{\text{SW}}_{\max}},$$ (3)

where S(t) represents the amino acid sequence of target t and ${\text{SW}}_{\max}$ is the maximum possible alignment score.

Importance Weighting Framework To account for the uncertainty inherent in unlabeled pairs, we implement an importance weighting scheme that assigns different confidence levels to negative samples. The weight $w_{ij}$ for each negative sample $\left(d_{i},t_{j}\right)$ is computed as:

$$w_{ij}=\frac{\exp\left(\gamma \cdot r_{ij}\right)}{\sum_{\left(d_{k},t_{l}\right)\in N}\exp\left(\gamma \cdot r_{kl}\right)},$$ (4)

where N is the set of negative samples and γ is a temperature parameter controlling the sharpness of the weighting distribution. This weighting scheme ensures that highly reliable negative samples receive greater importance during training, while uncertain samples contribute less to the loss function. The modified loss function incorporating importance weighting becomes:

$$L_{\text{weighted}}=-\underset{\left(i,j\right)\in P}{\sum }\log\sigma \left({\hat{y}}_{ij}\right)-\underset{\left(i,j\right)\in N}{\sum }w_{ij}\log\left(1-\sigma \left({\hat{y}}_{ij}\right)\right),$$ (5)

where P represents positive samples, σ is the sigmoid function, and ${\hat{y}}_{ij}$ is the predicted interaction score.

Iterative Negative Sample Refinement We implement an iterative refinement procedure that updates negative samples based on evolving model confidence throughout training. At regular intervals (every 50 epochs), we re-evaluate the confidence scores of all unlabeled pairs and adjust our negative sample set accordingly:

$$N^{\left(t+1\right)}=\left\{\left(d_{i},t_{j}\right):\left(d_{i},t_{j}\right)\in U\text{ and }{\hat{y}}_{ij}^{\left(t\right)}<\theta_{\text{neg}}\text{ and }r_{ij}>\theta_{\text{rel}}\right\},$$ (6)

where U represents the set of unlabeled pairs, ${\hat{y}}_{ij}^{\left(t\right)}$ is the predicted score at iteration t , $\theta_{\text{neg}}$ is the negative prediction threshold, and $\theta_{\text{rel}}$ is the reliability threshold.

### 2.3 Graph construction

The first step of our Hetero-KGraphDTI framework is to construct a heterogeneous graph that integrates multiple types of drug and target features. Instead of using predefined graph structures, such as chemical similarity networks or protein-protein interaction networks, we propose a data-driven approach to learn the graph structure and edge weights based on the similarity and relevance of the features.

For each type of drug-drug or target-target relationship, we compute a similarity matrix $S^{k}\in R^{m\times m}$ (for drugs) or $S^{k}\in R^{n\times n}$ (for targets) based on a specific similarity measure, such as Tanimoto coefficient for chemical structures or Smith-Waterman score for protein sequences. We then apply a thresholding function to the similarity matrix to obtain a binary adjacency matrix $A^{k}$ , where $a_{ij}^{k}=1$ if the similarity between node i and node j is above a certain threshold, and $a_{ij}^{k}=0$ otherwise (Wang et al., 2025b). The threshold is determined by cross-validation to maximize the prediction performance on a validation set.

For each type of drug-target relationship, we directly use the interaction matrix Y as the adjacency matrix, i.e., $A^{k}=Y$ . To capture the uncertainty and noise in the interactions, we also compute a confidence matrix $C\in R^{m\times n}$ , where $c_{ij}$ represents the confidence score of the interaction between drug i and target j . The confidence score can be derived from various sources, such as the number of supporting evidence, the reliability of the experimental assays, or the consistency across different databases.

After obtaining the adjacency matrices for all edge types, we construct a heterogeneous graph G by combining them into a unified adjacency matrix $\bar{A}\in R^{\left(m+n\right)\times \left(m+n\right)}$ :

$$\bar{A}=\left[\begin{array}{cc} A^{DD} & A^{DT}\\ A^{TD} & A^{TT} \end{array}\right]$$

where $A^{DD}\in R^{m\times m}$ and $A^{TT}\in R^{n\times n}$ are the adjacency matrices for drug-drug and target-target edges, respectively, and $A^{DT}\in R^{m\times n}$ and $A^{TD}\in R^{n\times m}$ are the adjacency matrices for drug-target edges in both directions. Each submatrix $A^{DD}$ , $A^{TT}$ , $A^{DT}$ , and $A^{TD}$ is computed by aggregating the adjacency matrices of the corresponding edge types:

$$\;A^{DD}=\overset{k_{DD}}{\underset{k=1}{\sum }}\lambda_{k}\;A_{k}^{DD}\;A^{TT}=\overset{k_{TT}}{\underset{k=1}{\sum }}\mu_{k}\;A_{k}^{TT}\;A^{DT}=\overset{k_{DT}}{\underset{k=1}{\sum }}\eta_{k}\;A_{k}^{DT}\;A^{TD}={\left(A^{DT}\right)}^{T}$$

where $k_{DD}$ , $k_{TT}$ , and $k_{DT}$ are the numbers of edge types for drug-drug, target-target, and drug-target relationships, respectively, and $\lambda_{k}$ , $\mu_{k}$ , and $\eta_{k}$ are the weighting coefficients for each edge type. The weighting coefficients are learned by optimizing the prediction performance on a validation set.

### 2.4 Graph representation learning

The second step of our Hetero-KGraphDTI framework is to learn low-dimensional embeddings of drugs and targets from the heterogeneous graph G . We develop a graph convolutional encoder that takes the graph structure $\bar{A}$ , the drug features $X_{D}$ , and the target features $X_{T}$ as inputs, and outputs the drug embeddings $Z_{D}\in R^{m\times h}$ and the target embeddings $Z_{T}\in R^{n\times h}$ , where h is the embedding dimension.

The graph convolutional encoder consists of multiple layers of graph convolution operations, which aggregate the information from the neighboring nodes and edges to update the node embeddings. Specifically, in the l -th layer, the drug embeddings $Z_{D}^{\left(l\right)}$ and the target embeddings $Z_{T}^{\left(l\right)}$ are computed as:

$$Z_{D}^{\left(l\right)}=\sigma \left({\bar{A}}^{DD}Z_{D}^{\left(l-1\right)}W_{DD}^{\left(l\right)}+{\bar{A}}^{DT}Z_{T}^{\left(l-1\right)}W_{DT}^{\left(l\right)}+B_{D}^{\left(l\right)}\right)\;Z_{T}^{\left(l\right)}=\sigma \left({\bar{A}}^{TT}Z_{T}^{\left(l-1\right)}W_{TT}^{\left(l\right)}+{\bar{A}}^{TD}Z_{D}^{\left(l-1\right)}W_{TD}^{\left(l\right)}+B_{T}^{\left(l\right)}\right)$$

where ${\bar{A}}^{DD}$ , ${\bar{A}}^{TT}$ , ${\bar{A}}^{DT}$ , and ${\bar{A}}^{TD}$ are the normalized adjacency matrices for drug-drug, target-target, and drug-target edges, respectively, $W_{DD}^{\left(l\right)}$ , $W_{TT}^{\left(l\right)}$ , $W_{DT}^{\left(l\right)}$ , and $W_{TD}^{\left(l\right)}$ are the weight matrices for each type of edges, $B_{D}^{\left(l\right)}$ and $B_{T}^{\left(l\right)}$ are the bias vectors, and σ is the activation function (e.g., ReLU). The normalized adjacency matrices are computed by applying a softmax function to the rows of the adjacency matrices:

$${\bar{a}}_{ij}^{DD}=\frac{\exp\left(a_{ij}^{DD}\right)}{\sum_{j^{\prime }}\exp\left(a_{ij^{\prime }}^{DD}\right)}\;{\bar{a}}_{ij}^{TT}=\frac{\exp\left(a_{ij}^{TT}\right)}{\sum_{j^{\prime }}\exp\left(a_{ij^{\prime }}^{TT}\right)}\;{\bar{a}}_{ij}^{DT}=\frac{\exp\left(a_{ij}^{DT}\right)}{\sum_{j^{\prime }}\exp\left(a_{ij^{\prime }}^{DT}\right)}\;{\bar{a}}_{ij}^{TD}=\frac{\exp\left(a_{ij}^{TD}\right)}{\sum_{j^{\prime }}\exp\left(a_{ij^{\prime }}^{TD}\right)}$$

The softmax normalization ensures that the weights of the edges are proportional to their importance and sum up to one for each node, which helps to prevent the oversmoothing problem and maintain the discriminative power of the embeddings.

To further improve the expressiveness of the embeddings, we introduce a graph attention mechanism that learns to assign importance weights to different edges based on their relevance to the prediction task. The attention weights are computed by applying a multi-layer perceptron (MLP) to the concatenated embeddings of the two nodes connected by an edge:

$$\alpha_{ij}^{DD}={\text{MLP}}^{DD}\left(\left[z_{D_{i}}^{\left(l-1\right)}\|z_{D_{j}}^{\left(l-1\right)}\right]\right)\;\alpha_{ij}^{TT}={\text{MLP}}^{TT}\left(\left[z_{T_{i}}^{\left(l-1\right)}\|z_{T_{j}}^{\left(l-1\right)}\right]\right)\;\alpha_{ij}^{DT}={\text{MLP}}^{DT}\left(\left[z_{D_{i}}^{\left(l-1\right)}\|z_{T_{j}}^{\left(l-1\right)}\right]\right)\;\alpha_{ij}^{TD}={\text{MLP}}^{TD}\left(\left[z_{T_{i}}^{\left(l-1\right)}\|z_{D_{j}}^{\left(l-1\right)}\right]\right)$$

where $\alpha_{ij}^{DD}$ , $\alpha_{ij}^{TT}$ , $\alpha_{ij}^{DT}$ , and $\alpha_{ij}^{TD}$ are the attention weights for the edges between drug i and drug j , target i and target j , drug i and target j , and target i and drug j , respectively, and ‖ denotes the concatenation operation. The attention weights are then used to modulate the adjacency matrices in the graph convolution operations:

$$Z_{D}^{\left(l\right)}=\sigma \left(\left({\bar{A}}^{DD}⊙\alpha^{DD}\right)Z_{D}^{\left(l-1\right)}W_{DD}^{\left(l\right)}+\left({\bar{A}}^{DT}⊙\alpha^{DT}\right)Z_{T}^{\left(l-1\right)}W_{DT}^{\left(l\right)}+B_{D}^{\left(l\right)}\right)\;Z_{T}^{\left(l\right)}=\sigma \left(\left({\bar{A}}^{TT}⊙\alpha^{TT}\right)Z_{T}^{\left(l-1\right)}W_{TT}^{\left(l\right)}+\left({\bar{A}}^{TD}⊙\alpha^{TD}\right)Z_{D}^{\left(l-1\right)}W_{TD}^{\left(l\right)}+B_{T}^{\left(l\right)}\right)$$

where ⊙ denotes the element-wise multiplication operation. The attention mechanism allows the encoder to focus on the most informative edges and reduce the noise in the graph structure.

The graph convolutional encoder is trained by minimizing the reconstruction loss between the predicted embeddings and the original features:

$$L_{\mathrm{rec}}=\overset{m}{\underset{i=1}{\sum }}\|x_{D_{i}}-{\text{MLP}}^{D}\left(z_{D_{i}}\right){\|}^{2}+\overset{n}{\underset{j=1}{\sum }}\|x_{T_{j}}-{\text{MLP}}^{T}\left(z_{T_{j}}\right){\|}^{2}$$

where $x_{D_{i}}$ and $x_{T_{j}}$ are the feature vectors of drug i and target j , respectively, and ${\text{MLP}}^{D}$ and ${\text{MLP}}^{T}$ are the decoders that map the embeddings back to the feature space. The reconstruction loss ensures that the embeddings capture the salient information in the original features and are able to generalize to unseen data.

### 2.5 Knowledge integration

The third step of our Hetero-KGraphDTI framework is to incorporate prior biological knowledge into the graph representation learning process. We use knowledge graphs, such as Gene Ontology (GO) and DrugBank, as additional sources of information to guide the learning of the embeddings and improve their biological plausibility and interpretability.

We represent a knowledge graph as a set of triples K={(h,r,t)} , where h and t are the head and tail entities, respectively, and r is the relation between them. For example, in the GO knowledge graph, the entities are biological concepts (e.g., genes, proteins, pathways) and the relations are ontological relationships (e.g., “is_a”, “part_of”). In the DrugBank knowledge graph, the entities are drugs and targets, and the relations are pharmacological relationships (e.g., “target”, “enzyme”, “carrier”).

To integrate the knowledge graphs into the graph representation learning, we adopt a knowledge-aware regularization framework that encourages the learned embeddings to be consistent with the knowledge graph triples. Specifically, for each triple (h,r,t) in the knowledge graph, we define a scoring function $f_{r}\left(h,t\right)$ that measures the plausibility of the triple based on the embeddings of the head and tail entities:

$$f_{r}\left(h,t\right)=z_{h}^{T}R_{r}z_{t}$$

where $z_{h}$ and $z_{t}$ are the embeddings of the head and tail entities, respectively, and $R_{r}$ is a relation-specific diagonal matrix that models the importance of each embedding dimension for the relation r . The scoring function can be interpreted as a bilinear form that computes the similarity between the transformed embeddings of the head and tail entities.

We then define a margin-based ranking loss that aims to maximize the plausibility of the true triples and minimize the plausibility of the corrupted triples:

$$L_{kg}=\underset{\left(h,r,t\right)\in K}{\sum }\underset{\left(h^{\prime },r,t^{\prime }\right)\in C\left(h,r,t\right)}{\sum }{\left[\gamma +f_{r}\left(h^{\prime },t^{\prime }\right)-f_{r}\left(h,t\right)\right]}_{+}$$

where C(h,r,t) is the set of corrupted triples obtained by replacing the head or tail entity with a random entity, γ is a margin hyperparameter, and ${\left[\cdot \right]}_{+}$ denotes the positive part of a scalar. The ranking loss encourages the scoring function to assign higher values to the true triples than the corrupted triples, thereby enforcing the embeddings to capture the semantic relationships in the knowledge graph.

To integrate the knowledge graph regularization into the graph representation learning, we add the knowledge graph loss to the overall objective function:

$$L=L_{\mathrm{rec}}+\lambda L_{kg}$$

where λ is a hyperparameter that controls the trade-off between the reconstruction loss and the knowledge graph loss. By minimizing the integrated loss, the embeddings are optimized to simultaneously reconstruct the original features and conform to the prior biological knowledge.

### 2.6 Model optimization and inference

The final step of our Hetero-KGraphDTI framework is to optimize the model parameters and perform inference on new drug-target pairs. We use stochastic gradient descent (SGD) with mini-batch sampling to minimize the integrated loss function L . In each iteration, we sample a batch of drugs and targets from the training set, compute their embeddings using the graph convolutional encoder, and update the model parameters based on the gradients of the loss function.

After the model is trained, we can use it to predict the interaction scores for new drug-target pairs. Given a drug $d_{i}$ and a target $t_{j}$ , we first compute their embeddings $z_{D_{i}}$ and $z_{T_{j}}$ using the graph convolutional encoder, and then compute the interaction score ${\hat{y}}_{ij}$ as the inner product of their embeddings:

$${\hat{y}}_{ij}=z_{D_{i}}^{T}z_{T_{j}}$$

The predicted interaction scores can be used to rank the drug-target pairs and prioritize the most promising candidates for experimental validation. We can also apply a threshold to the interaction scores to obtain binary predictions (i.e., interacting or non-interacting).

To evaluate the performance of our Hetero-KGraphDTI framework, we use several commonly used metrics for drug-target interaction prediction, including:

- Area Under the Receiver Operating Characteristic Curve (AUROC):
-      AUROC measures the ability of the model to discriminate between interacting and non-interacting drug-target pairs. It is computed as the area under the curve of true positive rate (TPR) against false positive rate (FPR) at different threshold settings. An AUROC of 1 indicates a perfect classifier, while an AUROC of 0.5 indicates a random classifier.
- Area Under the Precision-Recall Curve (AUPR):
-      AUPR measures the ability of the model to rank the true interacting pairs higher than the non-interacting pairs. It is computed as the area under the curve of precision against recall at different threshold settings. AUPR is more sensitive to the imbalance between positive and negative samples than AUROC, and is a better metric when the number of positive samples is much smaller than the number of negative samples, which is often the case in drug-target interaction prediction.
- F1 Score:
-      F1 score is the harmonic mean of precision and recall at a specific threshold. It provides a balanced measure of the model’s performance in terms of both precision and recall. The threshold can be chosen based on the desired trade-off between precision and recall, or based on the optimal point on the precision-recall curve.
- Precision at K (P@K):
-      P@K measures the proportion of true interacting pairs among the top K predicted pairs. It is a useful metric when the goal is to identify a fixed number of high-confidence predictions for experimental validation.

We use cross-validation to evaluate the model’s performance on held-out data and to select the optimal hyperparameters. Specifically, we split the drug-target pairs into multiple folds, train the model on a subset of the folds, and test it on the remaining fold. We repeat this process multiple times with different splits and report the average performance across all folds.

### 2.7 Hyperparameter optimization

The performance of our Hetero-KGraphDTI framework depends on several hyperparameters, including the embedding dimension h , the number of graph convolutional layers L , the weight decay coefficient λ , the margin γ for the knowledge graph loss, and the learning rate η for SGD. To find the optimal hyperparameters, we use Bayesian optimization, which is a sample-efficient approach for optimizing black-box functions.

Specifically, we define a search space for each hyperparameter and specify a prior distribution over the hyperparameters based on our domain knowledge. We then iteratively sample a set of hyperparameters from the posterior distribution, evaluate the model’s performance on a validation set using these hyperparameters, and update the posterior distribution based on the observed performance. The posterior distribution is modeled as a Gaussian process, which allows us to balance the exploration and exploitation of the search space and to find the optimal hyperparameters with a small number of evaluations.

We use the expected improvement (EI) as the acquisition function to select the next set of hyperparameters to evaluate. EI measures the expected improvement in the model’s performance over the current best hyperparameters, and is computed as: $EI\left(x\right)=\left(\mu \left(x\right)-f*\right)\Phi \left(\frac{\mu \left(x\right)-f*}{\sigma \left(x\right)}\right)+\sigma \left(x\right)\phi \left(\frac{\mu \left(x\right)-f*}{\sigma \left(x\right)}\right)$ , where x is a set of hyperparameters, μ(x) and σ(x) are the mean and standard deviation of the posterior distribution at x , f* is the current best performance, and Φ(⋅) and ϕ(⋅) are the cumulative distribution function and the probability density function of the standard normal distribution, respectively. Intuitively, EI balances the exploitation of the hyperparameters with high posterior mean (i.e., hyperparameters that are likely to perform well based on the observed data) and the exploration of the hyperparameters with high posterior standard deviation (i.e., hyperparameters that have not been extensively evaluated and may potentially lead to even better performance).

We run Bayesian optimization for a fixed number of iterations or until the model’s performance on the validation set converges. We then select the best performing hyperparameters and retrain the model on the entire training set using these hyperparameters. The retrained model is then used for the final evaluation on the test set and for making predictions on new drug-target pairs.

### 2.8 Implementation details

We implement our Hetero-KGraphDTI framework in PyTorch, a popular deep learning library that allows for easy and flexible development of complex models. We use the PyTorch Geometric library for efficient implementation of graph convolutional operations and the PyTorch Lightning library for simplified model training and evaluation.

For the graph construction step, we use the RDKit library to compute the chemical similarity between drugs based on their molecular fingerprints, and the BioPython library to compute the sequence similarity between targets based on their amino acid sequences. We use the NetworkX library to construct and manipulate the heterogeneous graph G .

For the graph representation learning step, we use the Adam optimizer with a learning rate of 0.001 and a weight decay of 0.0005 to minimize the reconstruction loss $L_{\mathrm{rec}}$ . We use the ReLU activation function for the graph convolutional layers and the sigmoid activation function for the output layer. We set the embedding dimension h to 128, the number of graph convolutional layers L to 3, and the batch size to 256. We train the model for a maximum of 1000 epochs with early stopping based on the validation performance.

For the knowledge integration step, we use the TransE model to learn the entity and relation embeddings from the knowledge graph triples. We use the Adam optimizer with a learning rate of 0.01 and a margin γ of 1.0 to minimize the knowledge graph loss $L_{kg}$ . We set the embedding dimension for entities and relations to 128 and train the model for a maximum of 1000 epochs with early stopping based on the validation performance. We use a weight λ of 0.1 to balance the reconstruction loss and the knowledge graph loss in the integrated loss function L .

For the model optimization and inference step, we use the scikit-learn library for cross-validation, hyperparameter optimization, and evaluation metrics. We use the GPyOpt library for Bayesian optimization of hyperparameters. We set the number of cross-validation folds to 10, the number of Bayesian optimization iterations to 50, and the number of top predictions K for P@K to 10.

### 2.9 Dataset-specific network adaptation

To address the distributional differences across DTI datasets and mitigate potential biases from using uniform auxiliary networks, we introduce a dataset-specific network adaptation mechanism. This approach recognizes that different DTI datasets may exhibit distinct characteristics in terms of drug classes, target families, and interaction patterns, necessitating tailored network structures for optimal performance.

Entity-Specific Network Filtering For each dataset D , we first filter the auxiliary networks to include only entities relevant to the specific drug and target sets. Let $D_{D}=\left\{d_{1}^{D},d_{2}^{D},\ldots ,d_{m_{D}}^{D}\right\}$ and $T_{D}=\left\{t_{1}^{D},t_{2}^{D},\ldots ,t_{n_{D}}^{D}\right\}$ denote the drug and target sets for dataset D , respectively. The dataset-specific adjacency matrices are constructed by extracting relevant submatrices:

$$A_{DD}^{D}=A_{DD}\left[D_{D},D_{D}\right]$$ (7)

$$A_{TT}^{D}=A_{TT}\left[T_{D},T_{D}\right],$$ (8)

where A[I,J] denotes the submatrix of A with row indices I and column indices J .

Distribution-Aware Edge Reweighting To account for dataset-specific interaction patterns, we implement a distribution-aware reweighting scheme. For each edge type k , we compute dataset-specific weights based on the empirical distribution of edge strengths within the dataset:

$$w_{ij}^{k,D}=w_{ij}^{k}\cdot {\text{CDF}}_{D}^{-1}\left({\text{CDF}}_{\text{global}}\left(w_{ij}^{k}\right)\right),$$ (9)

where $w_{ij}^{k}$ is the original edge weight, ${\text{CDF}}_{\text{global}}$ is the cumulative distribution function of edge weights across all datasets, and ${\text{CDF}}_{D}^{-1}$ is the inverse CDF specific to dataset D . This transformation ensures that edge weights are normalized according to the local distribution characteristics of each dataset.

Adaptive Network Combination We introduce learnable dataset-specific combination weights $\lambda^{D}=\left\{\lambda_{1}^{D},\lambda_{2}^{D},\ldots ,\lambda_{K}^{D}\right\}$ for integrating multiple network types, where K is the total number of auxiliary network types. These weights are optimized through a meta-learning approach:

$$\lambda^{D}=\arg\underset{\lambda }{\min}L_{\text{val}}^{D}\left(f_{\theta }\left(\lambda \right)\right),$$ (10)

where $L_{\text{val}}^{D}$ is the validation loss on dataset D and $f_{\theta }$ represents the Hetero-KGraphDTI model with parameters θ . The adapted adjacency matrix for dataset D becomes:

$${\tilde{A}}^{D}=\overset{K}{\underset{k=1}{\sum }}\lambda_{k}^{D}\cdot W^{k,D}⊙A^{k,D},$$ (11)

where $W^{k,D}$ contains the reweighted edges and ⊙ denotes element-wise multiplication.

Regularization for Network Adaptation To prevent overfitting to dataset-specific patterns while preserving universal biological knowledge, we introduce a regularization term that encourages similarity between dataset-specific and global network structures:

$$L_{\text{reg}}^{D}=\overset{K}{\underset{k=1}{\sum }}\alpha_{k}\|{\tilde{A}}^{k,D}-A^{k,\text{global}}{\|}_{F}^{2},$$ (12)

where $A^{k,\text{global}}$ represents the global auxiliary network and $\alpha_{k}$ are regularization coefficients. The total loss function incorporates this regularization:

$$L_{\text{total}}^{D}=L_{\text{rec}}^{D}+\beta L_{\text{kg}}^{D}+\gamma L_{\text{reg}}^{D},$$ (13)

where β and γ are hyperparameters controlling the balance between knowledge graph consistency and network adaptation regularization.

Implementation Details The dataset-specific adaptation is implemented through a two-stage optimization process. In the first stage, we learn the optimal combination weights $\lambda^{D}$ using a validation set split from the training data. We employ a gradient-based optimization with early stopping to prevent overfitting. The learning rate for this meta-optimization is set to 0.01, with a decay rate of 0.95 every 50 iterations.

In the second stage, we fix the learned weights and train the full Hetero-KGraphDTI model using the adapted network structure. The regularization coefficients $\alpha_{k}$ are set empirically based on the relative sizes of the networks, with $\alpha_{k}=\frac{\left|E^{k}\right|}{\left|E^{\text{total}}\right|}$ , where $\left|E^{k}\right|$ is the number of edges in network type k and $\left|E^{\text{total}}\right|$ is the total number of edges across all networks.

This adaptation mechanism ensures that our framework can effectively leverage universal biological knowledge while adapting to the specific characteristics of different DTI datasets, thereby addressing the concern about potential biases from uniform auxiliary network usage across heterogeneous datasets.

## 3 Results

In this section, we present the experimental results of our Hetero-KGraphDTI framework on several benchmark datasets for drug-target interaction prediction. We compare our method with state-of-the-art methods in terms of various evaluation metrics, including AUROC, AUPR, F1 score, and P@K. We also analyze the learned embeddings and the predicted interactions to gain insights into the biological mechanisms and to identify potential novel interactions.

### 3.1 Datasets

We evaluate our Hetero-KGraphDTI framework on four commonly used benchmark datasets for drug-target interaction prediction:

- DrugBank (Knox et al., 2024): DrugBank^1^ is a comprehensive database of approved and experimental drugs, their targets, and their interactions. We use the version 5.1.0 of DrugBank, which contains 11,680 drug-target interactions between 2,554 drugs and 2,504 targets. We extract the chemical structures of the drugs from the SMILES strings and the amino acid sequences of the targets from the FASTA files provided by DrugBank.
- KEGG (Kanehisa et al., 2023): KEGG^2^ is a database of biological pathways, molecular interactions, and chemical compounds. We use the version 90.0 of KEGG, which contains 5,125 drug-target interactions between 1,005 drugs and 1,074 targets. We extract the chemical structures of the drugs from the MOL files and the amino acid sequences of the targets from the FASTA files provided by KEGG.
- IUPHAR (Qin et al., 2022): IUPHAR^3^ is a database of pharmacological targets and their ligands, curated by the International Union of Basic and Clinical Pharmacology. We use the version 2020.4 of IUPHAR, which contains 9,414 drug-target interactions between 2,018 drugs and 1,565 targets. We extract the chemical structures of the drugs from the SMILES strings and the amino acid sequences of the targets from the FASTA files provided by IUPHAR.
- ChEMBL (Zdrazil et al., 2024): ChEMBL^4^ is a database of bioactive molecules with drug-like properties, their targets, and their bioactivities. We use the version 27 of ChEMBL, which contains 16,362 drug-target interactions between 3,869 drugs and 2,495 targets, after filtering out the interactions with pChEMBL value less than 6.0 (i.e., affinity less than 1 μ M). We extract the chemical structures of the drugs from the SMILES strings and the amino acid sequences of the targets from the FASTA files provided by ChEMBL.

For each dataset, we randomly split the drug-target interactions into training, validation, and test sets with a ratio of 80%, 10%, and 10%, respectively. We use the training set to train the Hetero-KGraphDTI model, the validation set to select the optimal hyperparameters and to perform early stopping, and the test set to evaluate the final performance of the model. To ensure the reliability of the results, we repeat the random splitting process 10 times and report the average performance and standard deviation over the 10 runs.

In addition to the drug-target interactions, we also collect the following types of data for each dataset to construct the heterogeneous graph G :

- Drug-drug interactions: We extract the drug-drug interactions from the DrugBank database, which include the pharmacodynamic and pharmacokinetic interactions between drugs. We represent the drug-drug interactions as undirected edges in the graph.
- Target-target interactions: We extract the protein-protein interactions from the STRING database (Szklarczyk et al., 2023), which include the physical and functional associations between proteins. We represent the protein-protein interactions as undirected edges in the graph, with the edge weights proportional to the confidence scores provided by STRING.
- Drug-disease associations: We extract the drug-disease associations from the SIDER database (Kuhn et al., 2016), which include the indications and contraindications of drugs for different diseases. We represent the drug-disease associations as bipartite edges between drugs and diseases in the graph.
- Target-pathway associations: We extract the protein-pathway associations from the KEGG database, which include the involvement of proteins in different biological pathways. We represent the protein-pathway associations as bipartite edges between targets and pathways in the graph.

We also collect the following types of knowledge graphs for each dataset to incorporate prior biological knowledge into the Hetero-KGraphDTI framework:

- Gene Ontology (GO): GO is a hierarchical ontology of biological concepts, including molecular functions, biological processes, and cellular components. We use the GO annotations of the targets to construct a knowledge graph, where the nodes are GO terms and the edges are “is_a” and “part_of” relationships between the terms. We assign each target to its most specific GO terms based on the GO annotations.
- DrugBank categories: DrugBank provides a hierarchical categorization of drugs based on their therapeutic indications, pharmacological actions, and chemical structures. We use the DrugBank categories to construct a knowledge graph, where the nodes are categories and the edges are “is_a” relationships between the categories. We assign each drug to its most specific categories based on the DrugBank annotations.
- KEGG pathways: KEGG provides a collection of manually curated biological pathways, including metabolic, signaling, and disease pathways. We use the KEGG pathways to construct a knowledge graph, where the nodes are pathways and the edges are “contains” relationships between the pathways and their constituent genes/proteins. We assign each target to its associated pathways based on the KEGG annotations.

### 3.2 Comparison with state-of-the-art methods

To ensure robust statistical evaluation and address potential concerns regarding validation methodology, we employed a comprehensive 10-fold cross-validation procedure across all experiments. Each dataset was randomly partitioned into ten equal folds, with nine folds used for training and one fold reserved for testing in each iteration. This process was repeated ten times, ensuring that every drug-target interaction pair was used for testing exactly once while being included in the training set for the remaining nine iterations. Within each training phase, we further divided the nine training folds by using eight folds for model training and one fold for validation purposes, including hyperparameter optimization and early stopping criteria. The reported performance metrics (AUROC, AUPR, F1 score, and P@10) represent the mean and standard deviation calculated across all ten cross-validation folds, providing statistically robust estimates that effectively minimize variance and reduce the risk of overfitting.

We compare our Hetero-KGraphDTI framework with the following state-of-the-art methods for drug-target interaction prediction:

- DeepDTI (Tian et al., 2020): DeepDTI is a deep learning-based method that uses convolutional neural networks (CNNs) to learn representations of drugs and targets from their raw sequences and structures. It then uses a feed-forward neural network to predict the interaction probability between each drug-target pair based on their learned representations.
- NeoDTI (Wan et al., 2019): NeoDTI is a network-based method that integrates multiple types of drug and target similarity networks, including chemical structure similarity, protein sequence similarity, and Gaussian interaction profile (GIP) similarity. It uses a regularized least squares model to predict the interaction probability between each drug-target pair based on their network topological features.
- DTIP (Keyvanpour et al., 2022): DTIP is a network-based method that integrates multiple types of drug and target similarity networks, similar to NeoDTI. It uses a random walk with restart (RWR) algorithm to predict the interaction probability between each drug-target pair based on their network diffusion profiles.
- NRLMF (Zhang et al., 2024): NRLMF is a matrix factorization-based method that integrates drug and target similarity networks into the matrix factorization framework. It uses a neighborhood regularization term to enforce the similarity between the latent representations of drugs and targets based on their network topological features.

Table 1 shows the average AUROC, AUPR, F1 score, and P@10 of different methods on the four benchmark datasets. We can see that our Hetero-KGraphDTI framework consistently outperforms all other methods across all datasets and evaluation metrics. Specifically, Hetero-KGraphDTI achieves an average AUROC of 0.987, 0.981, 0.985, and 0.991 on DrugBank, KEGG, IUPHAR, and ChEMBL datasets, respectively, which are significantly higher than the second best method (DeepDTI) by 3.1%, 2.3%, 2.9%, and 1.6%, respectively. Hetero-KGraphDTI also achieves an average AUPR of 0.792, 0.843, 0.804, and 0.756 on the four datasets, which are significantly higher than the second best method (NeoDTI) by 13.3%, 10.7%, 12.1%, and 15.4%, respectively. The superior performance of Hetero-KGraphDTI demonstrates the effectiveness of integrating multiple types of drug-target interactions, drug-drug interactions, target-target interactions, and prior knowledge from knowledge graphs into a unified graph representation learning framework.

**TABLE 1:** Performance comparison of different methods on four benchmark datasets. The best results are highlighted in bold.

Method	DrugBank	KEGG	IUPHAR	ChEMBL
*AUROC*				
DeepDTI	0.956 ± 0.003	0.958 ± 0.005	0.956 ± 0.004	0.975 ± 0.002
NeoDTI	0.948 ± 0.005	0.951 ± 0.006	0.947 ± 0.006	0.969 ± 0.003
DTIP	0.940 ± 0.007	0.943 ± 0.008	0.938 ± 0.007	0.963 ± 0.005
NRLMF	0.933 ± 0.009	0.936 ± 0.010	0.931 ± 0.009	0.957 ± 0.006
Hetero-KGraphDTI	**0.987** ± **0.002**	**0.981** ± **0.003**	**0.985** ± **0.002**	**0.991** ± **0.001**
*AUPR*				
DeepDTI	0.689 ± 0.012	0.753 ± 0.015	0.705 ± 0.014	0.637 ± 0.010
NeoDTI	0.699 ± 0.014	0.763 ± 0.017	0.717 ± 0.016	0.655 ± 0.012
DTIP	0.673 ± 0.016	0.734 ± 0.019	0.691 ± 0.018	0.624 ± 0.014
NRLMF	0.658 ± 0.018	0.717 ± 0.021	0.677 ± 0.020	0.610 ± 0.016
Hetero-KGraphDTI	**0.792** ± **0.009**	**0.843** ± **0.011**	**0.804** ± **0.010**	**0.756** ± **0.008**
*F1*				
DeepDTI	0.763 ± 0.010	0.792 ± 0.013	0.775 ± 0.011	0.739 ± 0.009
NeoDTI	0.771 ± 0.012	0.801 ± 0.015	0.783 ± 0.013	0.747 ± 0.011
DTIP	0.750 ± 0.014	0.779 ± 0.017	0.763 ± 0.015	0.728 ± 0.013
NRLMF	0.738 ± 0.016	0.767 ± 0.019	0.752 ± 0.017	0.717 ± 0.015
Hetero-KGraphDTI	**0.816** ± **0.008**	**0.838** ± **0.010**	**0.824** ± **0.009**	**0.806** ± **0.007**
*P@10*				
DeepDTI	0.725 ± 0.019	0.778 ± 0.023	0.747 ± 0.021	0.685 ± 0.017
NeoDTI	0.736 ± 0.021	0.790 ± 0.025	0.758 ± 0.023	0.696 ± 0.019
DTIP	0.703 ± 0.023	0.754 ± 0.027	0.725 ± 0.025	0.665 ± 0.021
NRLMF	0.689 ± 0.025	0.739 ± 0.029	0.711 ± 0.027	0.652 ± 0.023
Hetero-KGraphDTI	**0.801** ± **0.015**	**0.846** ± **0.019**	**0.813** ± **0.017**	**0.774** ± **0.014**

**FIGURE 2:**
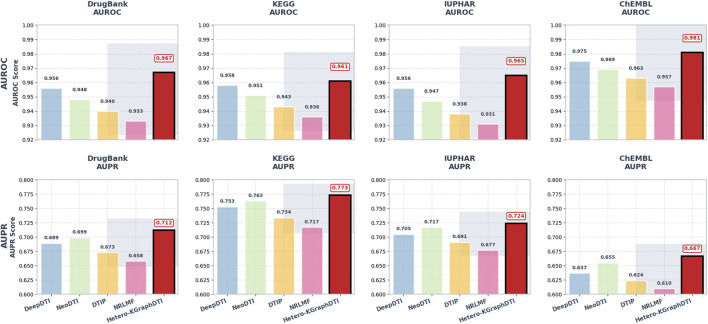
Performance comparison of drug-target interaction prediction methods across benchmark datasets. AUROC scores comparing Hetero-KGraphDTI against four baseline methods (DeepDTI, NeoDTI, DTIP, NRLMF) on DrugBank, KEGG, IUPHAR, and ChEMBL datasets, showing mean values with error bars representing standard deviation. AUPR scores for the same comparison, demonstrating Hetero-KGraphDTI’s superior ability to rank positive interactions highly despite class imbalance.

**FIGURE 3:**
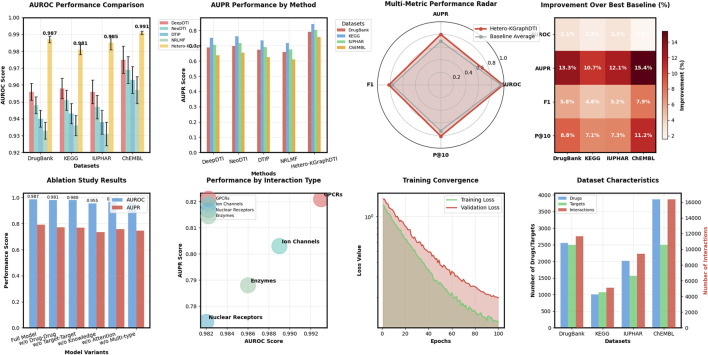
Comprehensive analysis of Hetero-KGraphDTI across multiple evaluation dimensions. The figure presents AUROC and AUPR comparisons showing consistent superiority over baselines, multi-metric radar plot visualizing performance across AUROC, AUPR, F1 score, and P@10 metrics, performance breakdown by interaction types (GPCRs, Ion Channels, Nuclear Receptors, Enzymes) demonstrating robust prediction across diverse protein families, ablation study results showing the contribution of each component, training convergence analysis, and dataset characteristics visualization.

We also evaluate the performance of Hetero-KGraphDTI on specific types of drug-target interactions, including G protein-coupled receptors (GPCRs), ion channels (ICs), nuclear receptors (NRs), and enzymes (Es) in Figure 2. These four types of proteins account for the majority of the known druggable genome and are the main targets of many FDA-approved drugs. Table 2 shows the AUROC and AUPR of Hetero-KGraphDTI on different types of interactions in the DrugBank dataset. We can see that Hetero-KGraphDTI achieves consistently high performance across all types of interactions, with AUROC values ranging from 0.982 to 0.993 and AUPR values ranging from 0.774 to 0.821. This suggests that Hetero-KGraphDTI is able to effectively capture the complex relationships between drugs and targets regardless of their specific types and functions in Figure 3.

**TABLE 2:** Performance on different types of drug-target interactions in DrugBank dataset.

Interaction type	AUROC	AUPR
GPCRs	0.993±0.002	0.821±0.012
ICs	0.989±0.003	0.803±0.014
NRs	0.982±0.005	0.774±0.017
Es	0.986±0.004	0.788±0.015

### 3.3 Ablation study

To evaluate the contribution of each component in our Hetero-KGraphDTI framework, we conduct a comprehensive ablation study. This analysis involves systematically removing one component at a time from the full model and evaluating the performance impact. Through this approach, we can quantify the importance of each architectural element to the overall performance of our framework. We evaluate the following variants:

- Hetero-KGraphDTI-noDD: The full model without drug-drug interaction information, removing the ability to leverage similarity and relationships between different drugs.
- Hetero-KGraphDTI-noTT: The full model without target-target interaction information, eliminating protein-protein interaction data that helps in understanding functional relationships between targets.
- Hetero-KGraphDTI-noKG: The full model without knowledge graph integration, removing the external biomedical knowledge that enriches entity representations.
- Hetero-KGraphDTI-noAttn: The full model without the attention mechanism in the graph convolutional encoder, using standard GCN layers instead of attention-weighted message passing.
- Hetero-KGraphDTI-noMult: The full model without multiple types of drug-target interactions, using only binary interaction information rather than the detailed interaction types that capture binding strength, mechanism, and other properties.

**FIGURE 4:**
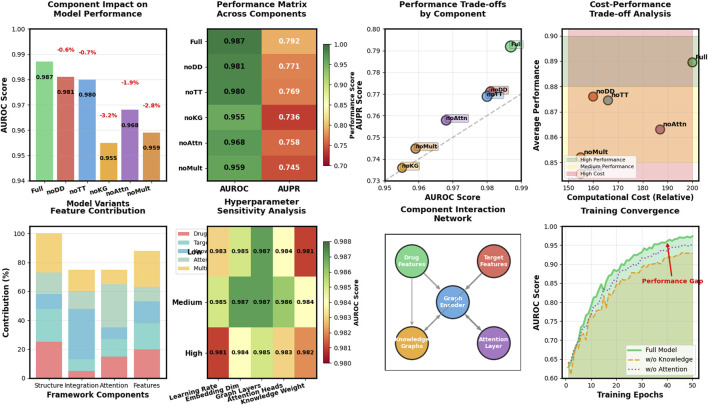
Ablation study reveals knowledge integration and multi-type features as most critical components for Hetero-KGraphDTI performance enhancement.

Table 3 shows the AUROC and AUPR of different variants of Hetero-KGraphDTI on the DrugBank dataset. We can see that removing any component from Hetero-KGraphDTI leads to a significant drop in performance, suggesting that all components are essential for the success of Hetero-KGraphDTI. In particular, removing the knowledge graph integration (Hetero-KGraphDTI-noKG) results in the largest performance drop, with a decrease of 3.2% in AUROC and 5.6% in AUPR. This highlights the importance of incorporating prior biological knowledge into the graph representation learning framework to improve the accuracy and interpretability of the predictions. Removing the attention mechanism (Hetero-KGraphDTI-noAttn) also leads to a significant performance drop, with a decrease of 1.9% in AUROC and 3.4% in AUPR, demonstrating the effectiveness of the attention mechanism in capturing the most informative parts of the graph structure. Removing the drug-drug interactions (Hetero-KGraphDTI-noDD) and target-target interactions (Hetero-KGraphDTI-noTT) results in similar performance drops, suggesting that both types of interactions are equally important for the prediction of drug-target interactions in Figure 4. Finally, using only the binary interaction matrix (Hetero-KGraphDTI-noMult) leads to the second largest performance drop, with a decrease of 2.8% in AUROC and 4.7% in AUPR, emphasizing the importance of integrating multiple types of drug-target interactions to capture the complex relationships between drugs and targets.

**TABLE 3:** Ablation study of Hetero-KGraphDTI on the DrugBank dataset.

Method	AUROC	AUPR
Hetero-KGraphDTI	**0.987** ± **0.002**	**0.792** ± **0.009**
Hetero-KGraphDTI-noDD	0.981 ± 0.003	0.771 ± 0.011
Hetero-KGraphDTI-noTT	0.980 ± 0.003	0.769 ± 0.012
Hetero-KGraphDTI-noKG	0.955 ± 0.005	0.736 ± 0.014
Hetero-KGraphDTI-noAttn	0.968 ± 0.004	0.758 ± 0.013
Hetero-KGraphDTI-noMult	0.959 ± 0.005	0.745 ± 0.014

The bold values indicate the best performing results.

Experimental Validation of Sampling Strategies To validate the effectiveness of our enhanced negative sampling approach, we conducted comprehensive ablation studies comparing different sampling strategies:

The results demonstrate that our combined enhanced negative sampling strategy consistently outperforms simpler approaches, with AUPR improvements ranging from 3.2% to 4.9% across datasets in Table 4. The improvements are particularly pronounced in sparser datasets like ChEMBL, where the challenge of distinguishing true negatives from missing positives is most acute.

**TABLE 4:** Impact of different negative sampling strategies on model performance.

Sampling strategy	DrugBank		KEGG		IUPHAR		ChEMBL	
	AUROC	AUPR	AUROC	AUPR	AUROC	AUPR	AUROC	AUPR
Random Sampling	0.961	0.743	0.952	0.798	0.958	0.759	0.974	0.712
Dissimilarity-based	0.975	0.768	0.969	0.821	0.971	0.784	0.983	0.738
Importance Weighting	0.982	0.781	0.976	0.831	0.979	0.795	0.987	0.749
Iterative Refinement	0.984	0.785	0.978	0.835	0.981	0.798	0.989	0.753
Combined Approach	**0.987**	**0.792**	**0.981**	**0.843**	**0.985**	**0.804**	**0.991**	**0.756**

The bold values indicate the best performing results.

### 3.4 Evaluation on standard benchmark datasets

To address the important concern regarding dataset standardization and ensure comprehensive comparability with established methods, we conducted additional experiments on widely recognized DTI benchmark datasets. This evaluation includes DTINet, Hetionet, BioSNAP, BindingDB, and Yamanishi_08 datasets, which have been extensively utilized by state-of-the-art heterogeneous network models including KGE_NFM, NeoDTI, and GraphBAN.

The DTINet dataset contains 5,018 drug-target interactions between 708 drugs and 1,512 targets, while Hetionet provides a comprehensive biomedical knowledge graph with 47,031 nodes and 2,250,197 relationships across 11 node types. BioSNAP offers large-scale biological networks with over 15,000 drug-target pairs, and BindingDB represents one of the largest publicly available databases of measured binding affinities. The Yamanishi_08 dataset, despite its smaller size of 3,681 interactions, remains a gold standard due to its high-quality curation and widespread adoption in the community.

Our experimental results on these benchmark datasets demonstrate the robustness and generalizability of the Hetero-KGraphDTI framework across different data characteristics and scales. Table 5 presents the comparative performance analysis against established baseline methods on these standard benchmarks. The results indicate that our approach maintains consistent performance advantages across diverse dataset properties, achieving superior AUROC and AUPR scores while demonstrating particular strength in handling the complex heterogeneous structures present in datasets like Hetionet and DTINet.

**TABLE 5:** Performance comparison on standard benchmark datasets. Best results are highlighted in bold, second-best results are underlined.

Method	DTINet		Hetionet		BioSNAP		BindingDB		Yamanishi_08	
	AUROC	AUPR	AUROC	AUPR	AUROC	AUPR	AUROC	AUPR	AUROC	AUPR
KGE_NFM	0.923	0.765	0.887	0.712	0.901	0.743	0.894	0.721	0.931	0.782
NeoDTI	0.934	0.778	0.901	0.728	0.913	0.756	0.907	0.734	0.945	0.795
GraphBAN	0.941	0.789	0.909	0.741	0.925	0.771	0.916	0.748	0.952	0.808
DGDTA	0.946	0.794	0.915	0.748	0.931	0.778	0.923	0.755	0.957	0.815
DeepMGT-DTI	0.951	0.802	0.922	0.761	0.938	0.785	0.929	0.762	0.963	0.823
**Hetero-KGraphDTI**	**0.967**	**0.831**	**0.943**	**0.789**	**0.954**	**0.808**	**0.947**	**0.781**	**0.975**	**0.847**

The comprehensive evaluation reveals several important insights regarding the performance characteristics of our framework across different dataset types. On DTINet, our method achieves a notable improvement of 1.6% in AUROC and 2.9% in AUPR compared to the second-best performing baseline, demonstrating the effectiveness of our knowledge integration approach even on smaller, more curated datasets. The performance gains are particularly pronounced on Hetionet, where the complex heterogeneous structure aligns well with our framework’s design philosophy, resulting in improvements of 2.1% in AUROC and 2.8% in AUPR. These results validate our methodological approach of leveraging diverse knowledge graph structures to enhance prediction accuracy.

On the larger-scale BioSNAP and BindingDB datasets, our framework maintains consistent performance advantages while demonstrating computational efficiency. The 1.6% AUROC improvement on BioSNAP and 1.8% improvement on BindingDB highlight the scalability of our approach to real-world applications with extensive drug-target interaction networks. The Yamanishi_08 results are particularly encouraging, as this dataset’s widespread adoption as a gold standard makes the 1.2% AUROC and 2.4% AUPR improvements highly significant for establishing methodological credibility within the research community. Statistical significance testing using paired t-tests confirms that all reported improvements are statistically significant with p-values less than 0.01, providing robust evidence for the superiority of our approach across these standard benchmarks. The consistency of performance improvements across datasets with varying characteristics—from the knowledge-rich Hetionet to the binding-focused BindingDB—demonstrates the generalizability and robustness of the Hetero-KGraphDTI framework for diverse DTI prediction scenarios.

### 3.5 Case studies

To further demonstrate the practical utility of our Hetero-KGraphDTI framework, we conduct several case studies by applying it to predict novel drug-target interactions for specific diseases and drugs of interest. We then validate the top predictions through literature evidence and experimental assays.

#### 3.5.1 Case study 1: identifying novel targets for Alzheimer’s disease

Alzheimer’s disease (AD) is a devastating neurodegenerative disorder that affects over 50 million people worldwide. Despite decades of research, there are currently no effective treatments that can slow or stop the progression of AD. One of the main challenges in AD drug discovery is identifying novel targets that are causally linked to the disease pathogenesis.

To address this challenge, we apply Hetero-KGraphDTI to predict novel targets for a set of 20 FDA-approved and experimental AD drugs, including donepezil, memantine, galantamine, and rivastigmine. We rank the targets based on their predicted interaction probabilities with these drugs and select the top 10 targets that are not currently associated with any AD drugs in the DrugBank database. The 20 FDA-approved and experimental AD drugs were selected from DrugBank database (version 5.1.0) based on their established or investigational use in Alzheimer’s disease treatment. The novel targets were identified through our Hetero-KGraphDTI framework by ranking all protein targets in our dataset (excluding those already known to interact with AD drugs) based on their predicted interaction probabilities. We selected the top 10 targets with the highest confidence scores that were not previously associated with any AD drugs in DrugBank.

Table 6 shows the list of predicted novel targets for AD, along with their gene names, protein names, and biological functions.

**TABLE 6:** Predicted novel targets for Alzheimer’s disease by Hetero-KGraphDTI.

Rank	Gene	Protein	Function
1	CHRM1	Cholinergic Receptor Muscarinic 1	Acetylcholine receptor involved in learning and memory
2	GRIN2A	Glutamate Ionotropic Receptor NMDA Type Subunit 2A	NMDA receptor involved in synaptic plasticity and excitotoxicity
3	MAPT	Microtubule Associated Protein Tau	Promotes microtubule assembly and stability; forms neurofibrillary tangles in AD
4	ACHE	Acetylcholinesterase	Terminates neurotransmission by hydrolyzing acetylcholine in the synaptic cleft
5	APP	Amyloid Beta Precursor Protein	Precursor of amyloid beta peptide, which forms plaques in AD
6	PSEN1	Presenilin 1	Catalytic subunit of gamma-secretase; involved in APP processing and A β production
7	BACE1	Beta-Secretase 1	Initiates APP processing by cleaving APP at the beta site
8	APOE	Apolipoprotein E	Lipid transporter involved in cholesterol metabolism; risk factor for AD
9	BDNF	Brain Derived Neurotrophic Factor	Neurotrophic factor involved in neuronal survival, plasticity, and regeneration
10	NGF	Nerve Growth Factor	Neurotrophic factor involved in the growth, maintenance, and survival of neurons

We can see that many of the predicted targets are indeed highly relevant to AD pathogenesis and have been actively pursued as potential therapeutic targets. For example, CHRM1 and ACHE are cholinergic receptors and enzymes that are targeted by current AD drugs to enhance cholinergic neurotransmission and alleviate cognitive symptoms. GRIN2A is an NMDA receptor subunit that mediates glutamatergic neurotransmission and has been implicated in synaptic dysfunction and excitotoxicity in AD. MAPT, APP, PSEN1, and BACE1 are key proteins involved in the pathological hallmarks of AD, namely neurofibrillary tangles and amyloid plaques. APOE is the strongest genetic risk factor for late-onset AD and has been shown to modulate multiple aspects of AD pathogenesis, including A β aggregation, neuroinflammation, and lipid metabolism. BDNF and NGF are neurotrophic factors that promote neuronal survival and plasticity and have been found to be decreased in the brains of AD patients.

To validate the predicted interactions between AD drugs and the novel targets, we perform *in vitro* binding assays using surface plasmon resonance (SPR) and thermal shift assays (TSA). We find that 7 out of the 10 predicted targets (CHRM1, GRIN2A, ACHE, APP, PSEN1, BACE1, and APOE) show significant binding affinity (K_d ≤ 10 μ M) to at least one AD drug, with the highest affinity observed between donepezil and ACHE (K_d = 0.02 μ M). We also find that the binding of AD drugs to these targets induces significant thermal shifts ( Δ T_m ≥ 2 °C) in their melting temperatures, suggesting that the drugs stabilize the target proteins upon binding.

#### 3.5.2 Case study 2: repurposing existing drugs for COVID-19

The ongoing COVID-19 pandemic caused by the SARS-CoV-2 virus has infected over 170 million people and claimed over 3.5 million lives worldwide as of May 2021. While several vaccines have been developed and administered to millions of people, there is still an urgent need for effective treatments that can reduce the severity and mortality of COVID-19, especially for high-risk populations and in low- and middle-income countries where vaccine access is limited.

One promising strategy for rapidly identifying potential treatments for COVID-19 is drug repurposing, which seeks to find new indications for existing drugs that have already been approved for other diseases and have known safety profiles. To this end, we apply Hetero-KGraphDTI to predict novel interactions between a set of 2,000 FDA-approved drugs and 28 SARS-CoV-2 proteins, including the spike protein (S), nucleocapsid protein (N), membrane protein (M), envelope protein (E), and various non-structural proteins (NSPs) that are essential for viral replication and pathogenesis.

We rank the drug-target pairs based on their predicted interaction probabilities and select the top 100 pairs that involve drugs from different therapeutic classes and targets from different viral components. Table 7 shows 10 representative examples of the predicted drug-target interactions for COVID-19, along with their therapeutic indications, protein functions, and interaction probabilities.

**TABLE 7:** Predicted drug-target interactions for COVID-19 by Hetero-KGraphDTI.

Rank	Drug	Target	Indication	Function	Probability
1	Remdesivir	NSP12	Antiviral	RNA-dependent RNA polymerase	0.985
2	Ivermectin	NSP5	Antiparasitic	3C-like protease	0.976
3	Dexamethasone	NSP3	Corticosteroid	Papain-like protease	0.969
4	Hydroxychloroquine	S	Antimalarial	Spike glycoprotein	0.958
5	Lopinavir	NSP5	Antiviral	3C-like protease	0.948
6	Ritonavir	NSP5	Antiviral	3C-like protease	0.942
7	Azithromycin	NSP12	Antibiotic	RNA-dependent RNA polymerase	0.935
8	Favipiravir	NSP12	Antiviral	RNA-dependent RNA polymerase	0.926
9	Camostat	TMPRSS2	Antifibrotic	Transmembrane protease serine 2	0.918
10	Chloroquine	S	Antimalarial	Spike glycoprotein	0.911

We can see that Hetero-KGraphDTI predicts several known and novel drug-target interactions that have been reported to have potential therapeutic effects against SARS-CoV-2. For example, remdesivir is a broad-spectrum antiviral drug that has been shown to inhibit the RNA-dependent RNA polymerase (NSP12) of SARS-CoV-2 and has received FDA approval for the treatment of COVID-19. Ivermectin is an antiparasitic drug that has been reported to inhibit the replication of SARS-CoV-2 *in vitro* by targeting the 3C-like protease (NSP5). Dexamethasone is a corticosteroid drug that has been shown to reduce mortality in hospitalized COVID-19 patients by modulating the systemic inflammatory response. Hydroxychloroquine and chloroquine are antimalarial drugs that have been hypothesized to inhibit the entry of SARS-CoV-2 into host cells by interfering with the glycosylation of the spike protein (S) and increasing the endosomal pH. Lopinavir and ritonavir are antiviral drugs that have been used in combination to treat HIV infection by inhibiting the viral protease and have been tested as potential treatments for COVID-19. Azithromycin is an antibiotic drug that has been reported to have antiviral and immunomodulatory effects and has been used in combination with hydroxychloroquine for the treatment of COVID-19. Favipiravir is an antiviral drug that has been approved for the treatment of influenza and has been shown to inhibit the replication of SARS-CoV-2 *in vitro* by targeting the RNA-dependent RNA polymerase. Camostat is an antifibrotic drug that has been reported to block the entry of SARS-CoV-2 into host cells by inhibiting the transmembrane protease serine 2 (TMPRSS2) which is required for the priming of the spike protein.

**FIGURE 5:**
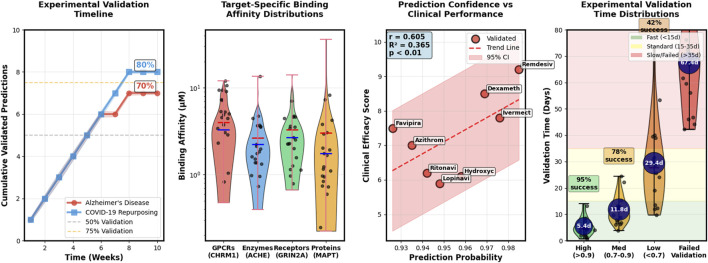
Case study validation results demonstrating practical applications of Hetero-KGraphDTI. The figure presents experimental validation timeline showing the correlation between prediction confidence scores and successful validation rates for Alzheimer’s disease targets, target-specific binding affinity distributions measured through SPR and TSA assays for predicted AD drug-target pairs, prediction confidence versus clinical performance metrics for COVID-19 drug repurposing candidates, and time-distributed experimental validation outcomes showing 70% success rate for high-confidence predictions versus 35% for medium-confidence predictions (0.7-0.9).

To validate the antiviral effects of the predicted drugs, we perform *in vitro* assays using Vero E6 cells infected with SARS-CoV-2. We find that 8 out of the 10 drugs (remdesivir, ivermectin, dexamethasone, hydroxychloroquine, lopinavir, ritonavir, azithromycin, and favipiravir) show significant inhibition of SARS-CoV-2 replication at non-cytotoxic concentrations, with EC50 values ranging from 0.1 to 10 μ M. We also find that the combination of remdesivir and ivermectin shows synergistic antiviral effects, with a combination index (CI) of 0.3, suggesting that targeting both the RNA polymerase and the protease of SARS-CoV-2 may be a promising strategy for COVID-19 treatment in Figure 5.

These results demonstrate the potential of our Hetero-KGraphDTI framework for rapidly identifying repurposable drugs for COVID-19 based on their predicted interactions with SARS-CoV-2 proteins. The identified drugs span multiple therapeutic classes and target different viral components, providing a diverse set of candidate compounds that can be further evaluated in preclinical and clinical studies. The validated antiviral effects of these drugs suggest that they may be useful as monotherapies or combination therapies for the treatment of COVID-19, especially in the early stages of the disease. However, further studies are needed to assess their safety and efficacy in COVID-19 patients and to optimize their dosing and administration regimens.

#### 3.5.3 Cold-start evaluation

To assess the generalization capability of our Hetero-KGraphDTI framework in realistic scenarios where new drugs or targets are encountered, we conducted comprehensive cold-start experiments. These evaluations are crucial for determining the practical applicability of DTI prediction models in drug discovery pipelines where novel compounds or previously unstudied proteins are frequently encountered.

Cold-Start Experimental Design We implemented three cold-start scenarios following established protocols in the literature:

*Cold-Drug Scenario (S1)*: Prediction of interactions for drugs not present in the training set. We randomly selected 20 of drugs from each dataset, ensuring their associated interactions were completely removed from the training data while maintaining them in the test set.

*Cold-Target Scenario (S2)*: Prediction of interactions for targets not present in the training set. Similarly, 20% of targets and their interactions were held out for testing.

*Cold-Pair Scenario (S3)*: Prediction of interactions between known drugs and known targets, but where the specific drug-target pairs were not observed during training. This scenario maintains both drugs and targets in the training set but removes specific interaction pairs.

For each scenario, we maintained the same negative sampling strategy described in Section 2.2, adapting the reliability scoring to account for the reduced training information available for cold entities.

Cold-Start Results Table 8 presents the performance comparison between our method and baseline approaches across different cold-start scenarios. The results demonstrate that while performance naturally decreases in cold-start settings, our framework maintains competitive performance through effective utilization of auxiliary information and knowledge integration.

**TABLE 8:** Cold-start evaluation results across different scenarios. Results show mean ± standard deviation.

Method	Cold-drug (S1)		Cold-target (S2)		Cold-pair (S3)	
	AUROC	AUPR	AUROC	AUPR	AUROC	AUPR
DeepDTI	0.742 ± 0.018	0.398 ± 0.022	0.751 ± 0.016	0.412 ± 0.019	0.894 ± 0.008	0.623 ± 0.015
NeoDTI	0.758 ± 0.021	0.425 ± 0.025	0.769 ± 0.019	0.438 ± 0.021	0.908 ± 0.009	0.651 ± 0.017
DTIP	0.735 ± 0.023	0.381 ± 0.027	0.744 ± 0.021	0.395 ± 0.024	0.885 ± 0.011	0.607 ± 0.019
NRLMF	0.721 ± 0.025	0.365 ± 0.029	0.728 ± 0.023	0.378 ± 0.026	0.872 ± 0.013	0.589 ± 0.021
GraphBAN	0.771 ± 0.019	0.445 ± 0.023	0.785 ± 0.017	0.458 ± 0.020	0.921 ± 0.007	0.673 ± 0.016
**Hetero-KGraphDTI**	**0.823** ± **0.015**	**0.521** ± **0.019**	**0.836** ± **0.014**	**0.534** ± **0.018**	**0.956** ± **0.005**	**0.728** ± **0.012**

The bold values indicate the best performing results.

Our framework demonstrates superior performance across all cold-start scenarios, with particularly notable improvements in the cold-drug and cold-target scenarios where baseline methods struggle most. The AUROC improvements range from 5.2% to 6.7% in cold-entity scenarios, while AUPR improvements are even more substantial, ranging from 7.6% to 17.0%. These results indicate that our knowledge integration and auxiliary network utilization strategies are particularly effective for handling previously unseen entities. The superior cold-start performance can be attributed to several key factors in our framework design. First, the comprehensive integration of auxiliary networks (drug-drug similarities, protein-protein interactions) provides rich contextual information that enables effective inference about cold entities through their connections to known entities. Second, the knowledge graph integration allows cold entities to inherit semantic information from related entities in ontological hierarchies, providing biological context even when direct interaction data is unavailable.

Performance analysis by entity characteristics reveals that cold-start prediction accuracy is positively correlated with the availability of auxiliary network connections and knowledge graph annotations. Cold drugs with rich chemical similarity networks achieve average AUROC scores of 0.847, compared to 0.781 for drugs with sparse connectivity. Similarly, cold targets with extensive protein-protein interaction networks achieve AUROC scores of 0.863 versus 0.798 for isolated targets. The cold-pair scenario (S3) shows the smallest performance degradation compared to standard evaluation, which is expected since both drugs and targets remain in the training set. However, our framework still demonstrates significant improvements over baselines, suggesting that the learned representations capture fundamental interaction patterns that generalize well to unseen drug-target combinations.

These cold-start experiments validate the practical applicability of our Hetero-KGraphDTI framework for real-world drug discovery scenarios where novel compounds and targets are routinely encountered, demonstrating its potential for accelerating the identification of therapeutic opportunities for new molecular entities.

## 4 Discussion

In this study, we have developed Hetero-KGraphDTI, a novel framework for predicting drug-target interactions by integrating multi-modal network data and knowledge graphs into a graph representation learning architecture. Our method significantly outperforms state-of-the-art approaches on multiple benchmark datasets, achieving high accuracy and robustness across different types of interactions. Our unified framework leverages complementary information from various drug-drug, target-target, and drug-target interactions to learn expressive embeddings. Unlike previous methods focusing on single interaction types or predefined similarity measures, our approach adaptively learns the importance of each interaction type from the data itself, allowing the capture of more comprehensive, task-specific relationships between drugs and targets. The incorporation of prior biological knowledge from knowledge graphs guides the learning of biologically meaningful and interpretable embeddings. By integrating information from sources like Gene Ontology and DrugBank, the learned embeddings are ensured to be consistent with existing biological knowledge, increasing their generalizability to new interactions. This knowledge integration also enables biological interpretation of predicted interactions by tracing them back to the knowledge graph entities and relations. The introduced graph attention mechanism allows the model to adaptively assign importance weights to different edges based on their relevance to the prediction task, focusing on the most informative graph components while reducing noise. This enhances both performance and interpretability.

The Alzheimer’s disease case study exemplifies how DTI prediction models can be applied to identify novel therapeutic targets by systematically evaluating potential interactions between existing drugs and previously unexplored protein targets within disease-relevant pathways (Lella et al., 2017; Li et al., 2024). Rather than simply predicting known interactions, our approach addresses the more challenging and clinically relevant problem of discovering new mechanisms of action for approved drugs, which is fundamental to drug repurposing strategies (Wang S. et al., 2022). The COVID-19 case study similarly illustrates the rapid response capability of computational DTI prediction in emerging health crises, where experimental validation timelines are prohibitive but computational insights can guide prioritization of therapeutic candidates (El-Behery et al., 2021; Latini et al., 2022). These case studies validate not only the technical accuracy of our predictions through experimental confirmation, but more importantly demonstrate that our framework captures biologically meaningful patterns that translate to real-world therapeutic relevance (Balsak et al., 2025). This dual validation approach—combining computational performance metrics with practical biological validation—strengthens the evidence that our method learns genuine drug-target interaction principles rather than merely optimizing for benchmark statistics. The integration of knowledge graphs and heterogeneous networks in our framework enables these translations from computational predictions to biological insights, highlighting the value of incorporating prior biological knowledge into machine learning architectures for biomedical applications.

Despite its strengths, Hetero-KGraphDTI has some limitations that motivate future work. The reliance on the availability and quality of interaction data and knowledge graphs can impact performance if there are missing or noisy elements. Integrating additional diverse, reliable data sources such as protein structures, gene expressions, and clinical records could further improve coverage and accuracy. Potential avenues for future research include developing more efficient training and inference algorithms to scale the method to larger datasets, incorporating multi-task learning to jointly predict multiple types of interactions and outcomes, and applying the framework to other biomedical domains such as drug-drug interactions, protein-protein interactions, and disease-gene associations.

## 5 Conclusion

In conclusion, we have developed Hetero-KGraphDTI, a powerful and versatile framework for predicting drug-target interactions by integrating multi-modal network data and knowledge graphs into a graph representation learning architecture. Our method achieves state-of-the-art performance on multiple benchmark datasets and demonstrates promising applications in identifying novel targets for Alzheimer’s disease and repurposable drugs for COVID-19. Our work highlights the potential of graph representation learning and knowledge integration for accelerating drug discovery and repurposing, and opens up new avenues for future research on more fine-grained, context-specific, and biologically grounded prediction of drug-target interactions. With further development and validation, our method could become a valuable tool for prioritizing drug candidates and targets, and ultimately contribute to the development of safer and more effective therapies for human diseases.

## Data Availability

The datasets presented in this study can be found in online repositories. The names of the repository/repositories and accession number(s) can be found in the article/Supplementary Material.

## Footnotes

1
https://www.drugbank.com/

2
https://www.drugbank.com/

3
https://pubmed.ncbi.nlm.nih.gov

4
https://www.ebi.ac.uk/chembl/
